# 开放性实验:基于超高效液相色谱-串联质谱法的甲状腺癌组织切片蛋白质组定量分析

**DOI:** 10.3724/SP.J.1123.2024.07009

**Published:** 2025-03-08

**Authors:** Yilan LI, Huiming YUAN, Jingtian CAO, Yidi ZHENG, Lan LI, Peifeng GAO

**Affiliations:** 1.北京理工大学分析测试中心, 北京 102488; 1. Analysis and Testing Center, Beijing Institute of Technology, Beijing 102488, China; 2.中国科学院大连化学物理研究所, 中国科学院分离分析化学重点实验室, 辽宁 大连 116023; 2. CAS Key Laboratoryof Separation Sciences for Analytical Chemistry, Dalian Institute of Chemical Physics, Chinese Academy of Sciences, Dalian 116023, China; 3.北京理工大学求是书院, 北京 102488; 3. Qiushi College, Beijing Institute of Technology, Beijing 102488, China; 4.北京理工大学北京书院, 北京 102488; 4. School of Beijing, Beijing Institute of Technology, Beijing 102488, China

**Keywords:** 液相色谱, 质谱, 蛋白质组学, 无标记定量, 实验教学, liquid chromatography (LC), mass spectrometry (MS), proteomics, label-free quantification, experimental teaching

## Abstract

开放性实验课程是培养本科生实践能力、创新思维和团队协作精神的重要手段。本项目基于超高效液相色谱-串联质谱法(UHPLC-MS/MS)在蛋白质组前沿领域的研究,设计了一个本科生开放性实验,即基于UHPLC-MS/MS完成甲状腺癌组织切片蛋白质组定量分析。首先,评价不同蛋白质裂解液对蛋白质的提取能力,以筛选适当的试剂用于蛋白质组样品预处理,可培养学生的动手能力和科研思维。其次,优化液相色谱-质谱采集方法,实现蛋白质组的深度覆盖分析,加深学生对于液相色谱-质谱技术的认识。最后,将该方法用于石蜡包埋甲状腺乳头状癌组织切片样品蛋白质组定量分析,发现了33个差异蛋白质,展示出在疾病分型方面的应用潜力。该开放性实验将仪器分析、分析化学和生物化学三门课程理论与实验相关知识交叉融合在一起,将科研思路和创新理念渗透给本科生。以进行大型仪器操作和创新性实验的形式帮助学生拓展巩固理论知识,有助于学生形成系统化知识体系,增强其对相关领域知识的整体把握,激发本科生科学研究的兴趣,开拓创新思维,增加团队合作精神。

## Abstract (en)

Open experiments play crucial roles in developing undergraduate students’ practical abilities, innovative thinking, and teamwork. This open experiment is designed to use ultra-high performance liquid chromatography-tandem mass spectrometry (UHPLC-MS/MS) in proteomics, a frontier field, by quantitatively analyzing thyroid-cancer-tissue-slice samples. Various reagents are first screened for further study by evaluating their abilities to extract proteins. This approach aims to foster students’ hands-on abilities and their scientific-research thinking. The liquid-chromatography method is subsequently optimized to enable deep-coverage of the proteome, thereby enhancing students’ understanding of liquid chromatography and mass spectrometry. Paraffin-embedded thyroid-cancer-tissue slices are finally subjected to quantitative proteomic analysis, resulting in the identification of 33 differentially expressed proteins, thereby demonstrating their potential use in disease-typing applications. This open experiment integrates theoretical and experimental knowledge gained through instrumental analysis, analytical chemistry, and biochemistry, thereby imparting scientific research thinking and innovative spirit to undergraduates. It also provides students with opportunities to expand and solidify theoretical knowledge through hands-on instrumental operation and experiments, which helps to build a systematic knowledge framework and develop a comprehensive understanding of relevant fields. Moreover, it stimulates undergraduate interest in scientific research, cultivates innovative thinking, and fosters team cooperation.

在当前高等教育体系中,培养学生的实践能力、创新思维和团队协作精神已成为教育的重要目标^[1,2]^。实验教学是将理论知识与实践操作相结合的关键环节。随着教育理念的不断更新和教学方法的多元化,开放实验课程作为一种新兴的教学模式,逐渐受到各个高校的关注^[3⇓-5]^。北京理工大学开放实验课程面向本科生,旨在打破传统实验教学的约束,赋予学生更多自主权,鼓励其参与科学研究,培养学生主动学习的能力,强化实践教学环节,以实现知识的内化与创新能力的培养。

超高效液相色谱-串联质谱法(UHPLC-MS/MS)结合了液相色谱的高效分离能力和质谱的高检测灵敏度和准确性,使得其对于复杂体系样品的分析具有显著优势,被广泛应用于食品安全、环境科学、生物医学等领域^[6,7]^。蛋白质是构成细胞、组织的重要结构和功能物质,是生命活动的基础。基于液相色谱-质谱技术实现临床样品蛋白质组分析对于疾病发生发展机制研究、潜在生物标志物发现以及疾病精准诊疗等具有重要意义^[8,9]^。福尔马林固定石蜡包埋的组织切片(FFPE)样品在临床上具有重要的研究价值,但是其中蛋白质含量低、组成复杂且基质干扰严重^[10]^。蛋白质组样品制备方法作为蛋白质组分析的前提,直接影响分析结果的准确度与可靠性^[11]^。本实验首先考察不同裂解液对蛋白质的提取能力,筛选适当的试剂完成蛋白质组样品的制备。由于液相色谱-质谱分析方法决定了蛋白质组鉴定的覆盖度,因此进一步优化了液相色谱分离条件。最终,将发展的方法用于甲状腺肿(goiter, G)和甲状腺乳头状癌(papillary thyroid carcinoma, T)FFPE蛋白质样品定量分析。

本课程理论教学部分涉及液相色谱和质谱发展历史、UHPLC-MS/MS分析原理、液相色谱-质谱的谱图解析、UHPLC-MS/MS在精准医学领域的应用等理论知识,利于提升学生的专业素养,拓展学生的知识视野。本课程实验部分涉及液相色谱-质谱联用仪使用、蛋白质组样品制备、蛋白质组样品数据分析等内容。将生物化学、分析化学、仪器分析三门课程知识交叉融合在一起,这种方式有助于学生形成系统化的知识体系,增强对相关领域知识的整体把握。同时,可以使学生更全面地理解分析化学和仪器分析在生物领域中的应用,进而形成完整的知识链条。此外,将实验操作、仪器分析与理论相结合,促使学生将理论知识应用到实际中,从而更深刻地理解和掌握知识,加深对科研的认识,激发学生探究未知领域的兴趣。

## 1 实验部分

### 1.1 仪器、试剂与材料

Ultimate 3000 RPLC高效液相色谱系统、Q Exactive HF-X高分辨质谱仪均购自Thermo Fisher公司;scientz-IID细胞超声破碎仪购自新芝生物公司。

十二烷基硫酸钠(SDS, ≥98.5%)、尿素(urea, ≥99%)、盐酸胍(GuHCl, ≥99%)、三羧基乙基膦(TCEP, ≥98.5%)、碘乙酰胺(IAA, ≥99%)、碳酸氢铵(≥99.5%)、甲酸(FA, ≥96%)、磷酸盐缓冲溶液(1×PBS)均购于Sigma-Aldrich公司,胰蛋白酶(质谱级)购自Promega公司,色谱纯乙腈购自Merck公司。

### 1.2 蛋白质组样品预处理

#### 1.2.1 细胞破碎

将Hela细胞分别重悬于200 μL的40 g/L SDS、6 mol/L盐酸胍、8 mol/L尿素或1×PBS缓冲液中,将样品放置于冰上。利用细胞超声破碎仪对细胞超声破碎。超声条件如下:5 s开,5 s关,反复操作,总时间1 min,能量40 W。在利用不同细胞裂解液破碎细胞时,用甲醇和去离子水交叉清洗超声探头3次,避免样品间的交叉污染。超声后的溶液在16000 g下离心20 min,去除沉降的不溶物,收集上清液即得到从细胞中提取的蛋白质溶液。

#### 1.2.2 蛋白质组样品预处理

在蛋白质溶液中加入终浓度为10 mmol/L的TCEP,混匀后在95 ℃反应10 min使蛋白质变性还原。放至室温后,加入终浓度为20 mmol/L的IAA,在室温避光反应30 min。采取基于滤膜辅助的蛋白质组样品预处理方法完成蛋白质组样品处理^[12]^。具体如下:将样品转移到截留分子质量为10000 Da的滤膜上,在16000 g下离心40 min。用200 μL 8 mol/L尿素和50 mmol/L碳酸氢铵溶液分别清洗滤膜3次。按照酶和蛋白质的质量比1∶20加入胰蛋白酶,在37 ℃下反应16 h。在16000 g下离心40 min回收酶解得到的肽段,用30 μL 50 mmol/L碳酸氢铵溶液清洗滤膜两次,离心后与肽段合并。冻干后用0.1%(v/v) FA水溶液复溶后冻存于-80 ℃,待UHPLC-MS/MS分析,以上各组实验均重复3次进行。

### 1.3 组织切片样品处理

甲状腺肿和甲状腺乳头状癌(T1N1M0)患者FFPE样品来源于大连医科大学附属第二医院,样品经过伦理委员会验证(大医二院伦快审2020第057号)。将切片样品放于50 mL烧杯中,加入二甲苯溶液浸没组织,共浸泡15 min。再分别用无水乙醇、70%(v/v)乙醇水溶液、56%(v/v)乙醇水溶液各浸泡组织两次,每次10 min,以完成石蜡包埋的组织切片样品的脱蜡水合。之后,用60 μL的40 g/L SDS提取蛋白质,并完成蛋白质组样品预处理。

### 1.4 UHPLC-MS/MS分析

流动相A相为含0.1%(v/v)FA的2%(v/v)乙腈水溶液,B相为含0.1%(v/v)FA的80%(v/v)乙腈水溶液。将肽段上样于Acclaim PePMap RSLC C18色谱柱(250 mm×75 μm, 2 μm)进行分离,流速为300 nL/min。

#### 1.4.1 液相色谱流动相梯度优化

方法一:总梯度洗脱时间为50 min。0~30 min, 5%B~30%B; 30~35 min, 30%B~50%B; 35~38 min, 50%B~95%B; 38~43 min, 95%B; 43~44 min, 95%B~5%B; 44~50 min, 5%B。

方法二:总梯度洗脱时间为85 min。0~60 min, 5%B~30%B; 60~70 min, 30%B~50%B; 70~71 min, 50%B~95%B; 71~75 min, 95%B; 75~76 min, 95%B~5%B; 76~85 min, 5%B。

方法三:总梯度洗脱时间为130 min。0~5 min, 5%B~8%B; 5~105 min, 8%B~30%B; 105~120 min, 30%B~50%B; 120~121 min, 50%B~90%B; 121~125 min, 90%B; 125~126 min, 90%B~5%B; 126~130 min, 5%B。

#### 1.4.2 质谱采集条件

在正离子数据依赖(DDA)模式下采集数据。一级质谱采集质量范围为350~2000 Da,分辨率为60000,自动增益控制(AGC)值为3×10^6^,最大离子注入时间设置为30 ms。母离子通过27%的碰撞能量进行碎裂,采集电荷为+2~+7价态肽段。二级质谱扫描在15000分辨率下进行,AGC设置为5×10^4^,分离窗口为*m/z* 1.6,动态排除时间设置为45 s。

### 1.5 数据分析

利用Proteome Discoverer(PD)软件(版本2.4)分析质谱采集的数据。采用2021年2月2日在uniprot库中下载的人源数据库与数据进行匹配。一级母离子和二级碎裂离子的质量兼容度分别为1.0×10^-5^(10 ppm)和2.0×10^-5^(20 ppm)。设置胰蛋白酶为特异性酶切,最多允许2个漏切位点。将蛋白质N-末端的乙酰化(+42.0150 Da)和甲硫氨酸的氧化(+15.9949 Da)设置为可变修饰,半胱氨酸上的烷基化(+71.0371 Da)设置为固定修饰。控制肽段和蛋白质鉴定假阳性率(FDR)≤ 1%。利用MaxQuant软件(版本1.6.5.0)对质谱采集的数据进行无标记定量分析。搜库条件与PD软件设置保持一致。

## 2 结果与讨论

### 2.1 蛋白质提取方法的优化

以HeLa细胞为样品,考察不同蛋白质提取试剂对蛋白质提取效率的影响。分别采用40 g/L SDS、6 mol/L盐酸胍、8 mol/L尿素、1×PBS缓冲液提取相同量HeLa细胞中的蛋白质,并采取基于滤膜辅助的蛋白质组样品预处理方法完成蛋白质组样品处理^[12]^,利用质谱仪对酶解的肽段重复采集3次,采集数据导入至PD软件中合并检索,得到不同蛋白质提取试剂处理下的鉴定结果。如图1所示,40 g/L SDS溶液提取的蛋白质鉴定数量最高,1×PBS缓冲液提取的蛋白质鉴定数量最低。两者相比,SDS溶液的蛋白质鉴定数量高出32.3%。此外,40 g/L SDS溶液提取条件下鉴定的蛋白质能覆盖到其他3种提取试剂的64.4%~82.2%。与其他3种提取试剂相比,40 g/L SDS溶液能单独鉴定775个蛋白质,经过基因本体论(GO)细胞组成分析发现,高达34.1%(264个)蛋白质定位为与膜相关的蛋白质。以上结果归因于SDS是一种强效且具有两亲性的表面活性剂,对蛋白质有更强的溶解能力,有利于鉴定到更多的蛋白质。因此,为了提高蛋白质的鉴定覆盖度,后续采用40 g/L SDS溶液完成蛋白质样品提取。

**图1:**
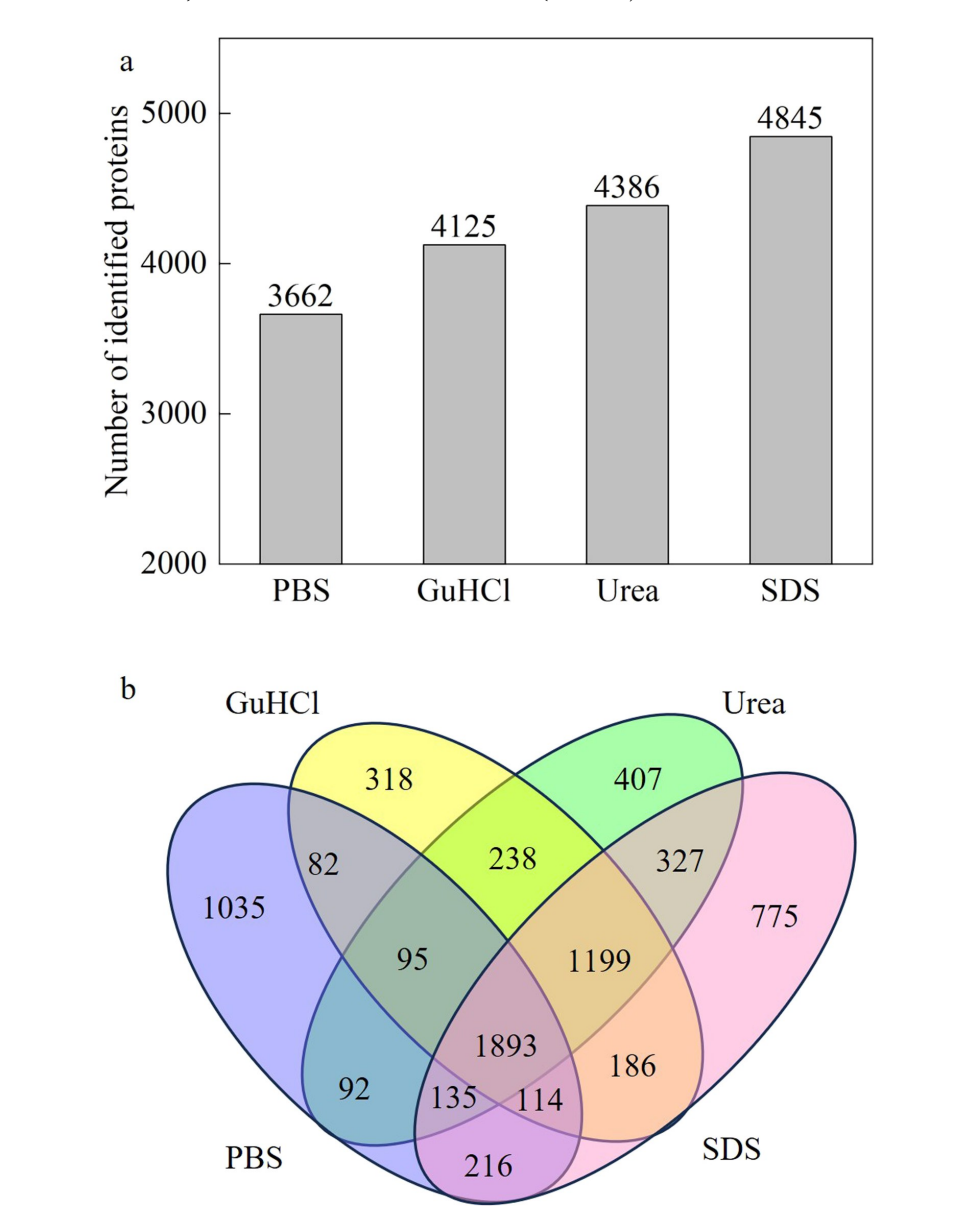
不同蛋白质提取试剂鉴定到的(a)蛋白质数量与 (b)蛋白质的韦恩图

### 2.2 UHPLC-MS/MS采集条件的优化

通过梯度洗脱方法,液相色谱可以有效地将复杂肽段混合物中的不同组分按照其保留性质的差异进行分离,进而被质谱检测。色谱分离梯度直接决定了多肽和蛋白质的鉴定覆盖度。因此,对液相色谱的梯度洗脱条件进行优化以评价相同样品在不同分离梯度下的色谱分离情况,并考察不同分离梯度对蛋白质鉴定覆盖度的影响。

用同一根色谱柱在50、85和130 min的分离梯度下分别分析Hela细胞酶解肽段。如图2所示,随着分离梯度时间的延长,肽段样品的出峰时间明显延长。此外,随机提取质量电荷比值(*m/z*)为855.46747的离子,如图3所示,在3种不同的分离梯度下,此离子的出峰时间分别为38.63、69.81和114.63 min,即同一组分在不同液相色谱条件下出峰时间不同。

**图2:**
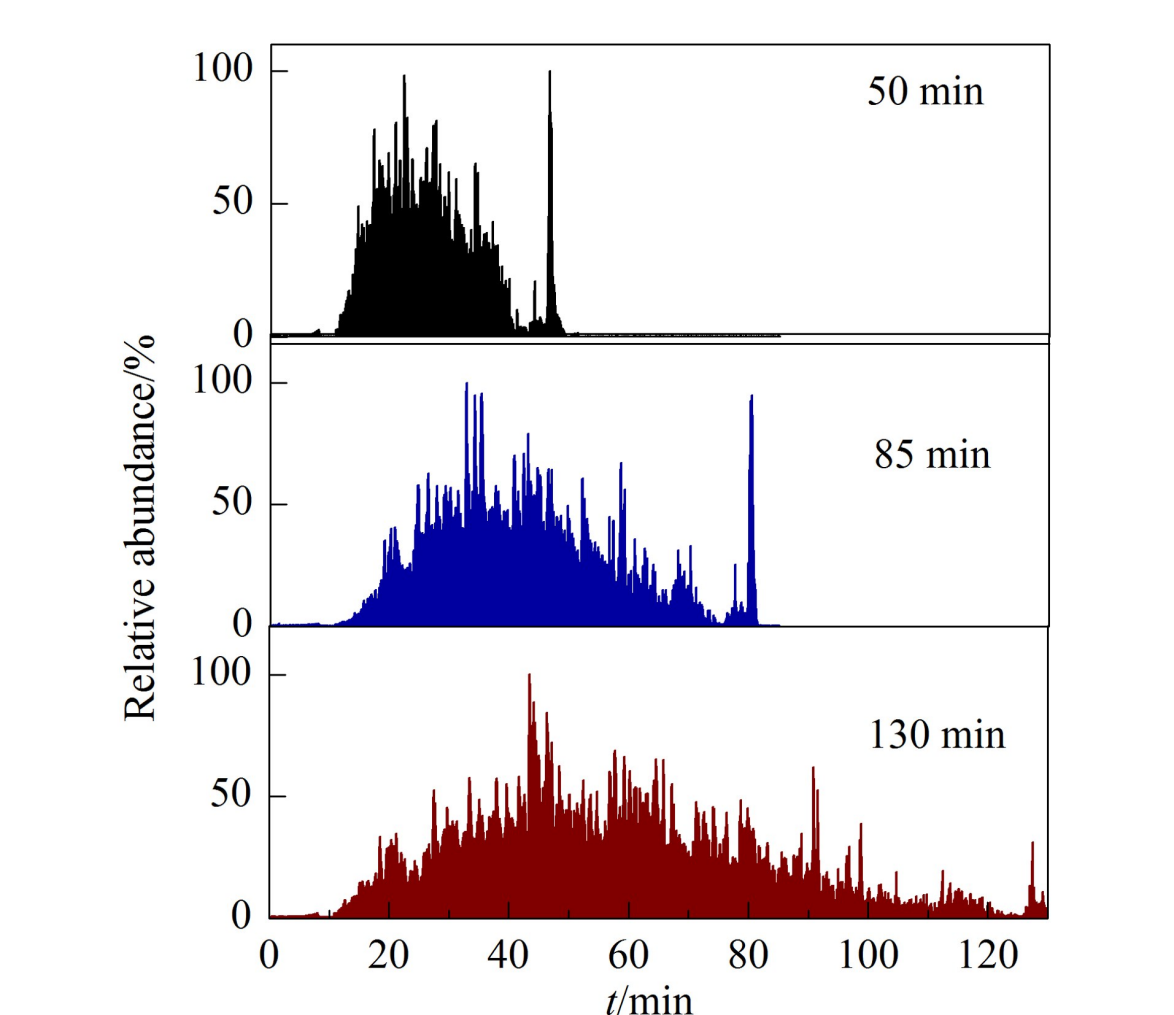
不同分离梯度下肽段的总离子流色谱图

**图3:**
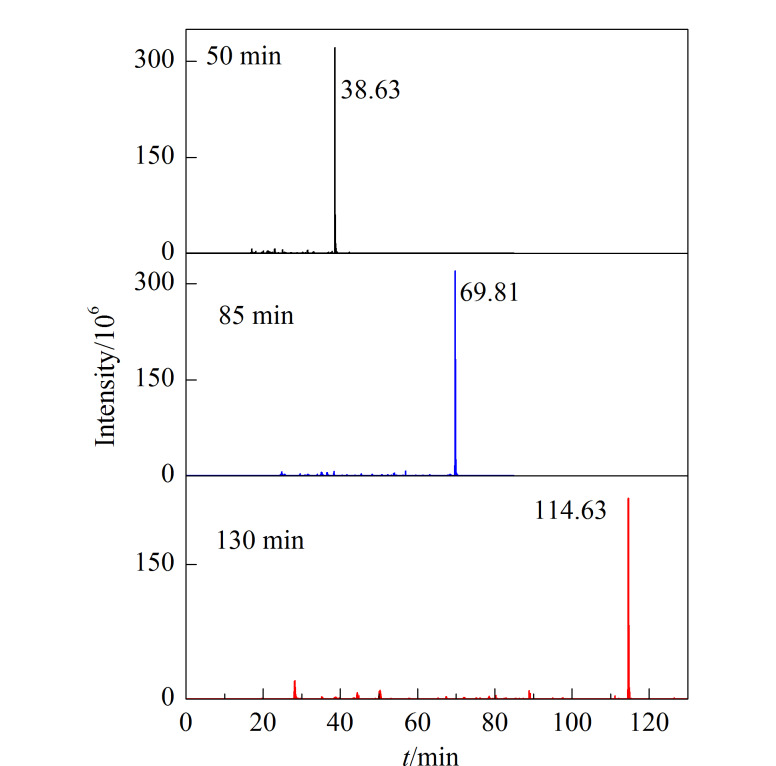
同一离子(*m/z* 855.46747)在不同分离梯度下 的提取离子色谱图

进一步对不同液相色谱分离梯度质谱鉴定的蛋白质进行分析。如图4a所示,在总分离时间为50、85和130 min的条件下,基于PD软件对每个条件下重复采集3针的数据合并检索。结果显示,蛋白质鉴定的数量分别为2767、3267、5029个,肽段的鉴定数量为7993、10034、24692条。即色谱分离梯度的延长实现了肽段更好的分离,利于蛋白质和肽段的鉴定。如图4b所示,130 min的分离梯度下鉴定的蛋白质可以覆盖50、85 min鉴定蛋白质的71.9%~72.5%。

**图4:**
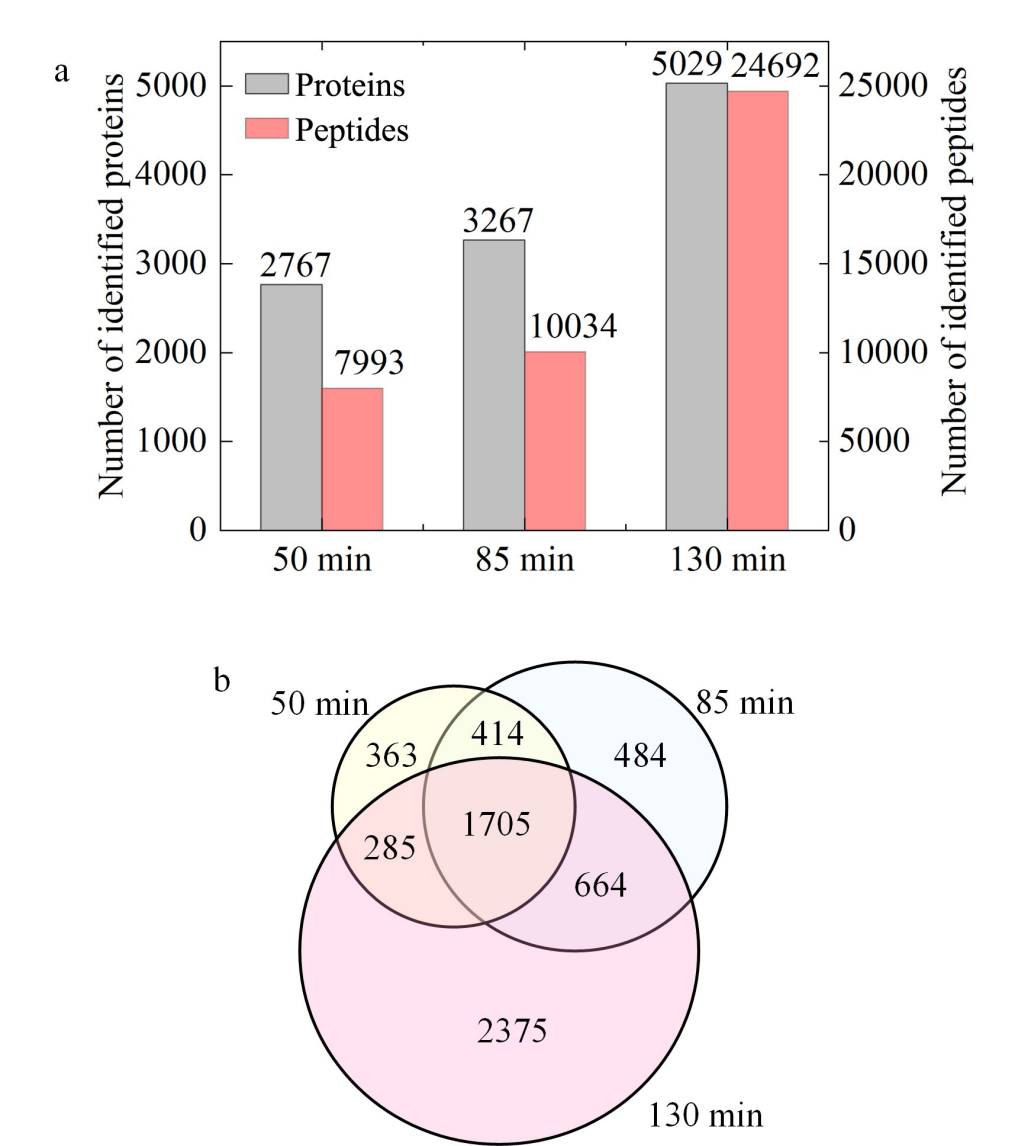
不同分离梯度下(a)蛋白质和肽段的鉴定数量与 (b)蛋白质的韦恩图

考虑到数据采集的重复性是准确定性定量分析的前提。因此,对数据采集的重复性进行评价。如图5a所示,在85 min的分离梯度下重复3次分析Hela细胞酶解肽段样品,同一离子(*m/z* 855.46747)在3针重复采集下的色谱保留时间有较好的一致性,保留时间的相对标准偏差只有0.04%。至少63.5%的蛋白质被3针重复鉴定,超过73.6%的蛋白质在至少两针中鉴定到(图5b)。比较3次质谱重复的定量比值,皮尔森相关系数为0.95~0.99(图5c),说明此方法具有较好的分析重复性。

**图5:**
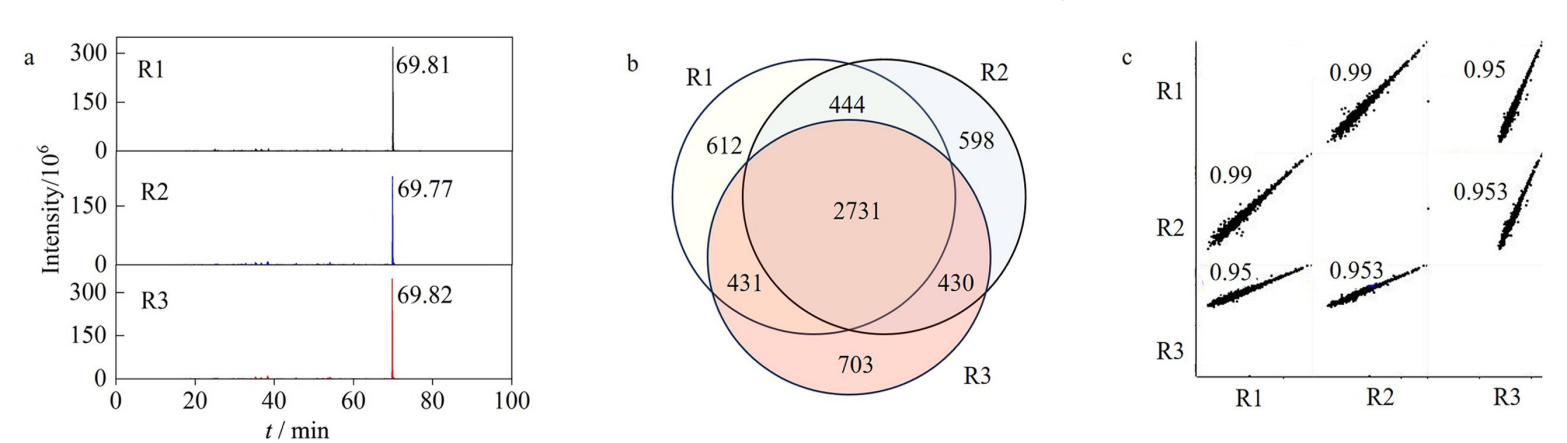
(a)在85 min的分离梯度下离子(*m/z* 855.46747)的色谱峰,(b)3次重复实验鉴定蛋白质的韦恩图及(c)峰强度的皮尔森相关性分析

### 2.3 甲状腺肿和甲状腺乳头状癌患者组织切片样本定量分析

FFPE样品在临床上具有重要的研究价值。甲状腺乳头状癌是一种在临床中常见的疾病。若不及时的干预和治疗,有进一步发展成晚期癌症和转移扩散的风险。因此,对甲状腺乳头状癌组织切片样品蛋白质组进行研究以期寻找疾病相关的潜在生物标志物,可助力疾病的及时发现和治疗,提高患者的治愈率和生存质量。

进一步对3例甲状腺肿和3例甲状腺乳头状癌的FFPE样品的蛋白质组进行分析。结果发现,两种组织切片样品中可以共同定量到432个蛋白质,任意两组样品之间的无标记定量强度的皮尔森相关系数超过0.85(图6),证明此方法具有良好的定量分析重复性。为保证分析的准确性和可信度,通过*t*检验,我们将*p*<0.05且甲状腺乳头状癌与甲状腺肿样品相比发生2倍变化的蛋白质作为差异蛋白。最终,与甲状腺肿相比,在甲状腺乳头状癌组织切片中定量到33个差异蛋白质,其中11个为上调蛋白质,22个为下调蛋白质(图7a),利用差异蛋白质成功实现了疾病的分子分型研究(图7b),证明利用组织切片蛋白质组可以成功实现甲状腺乳头状癌和甲状腺肿样品的区分。

**图6:**
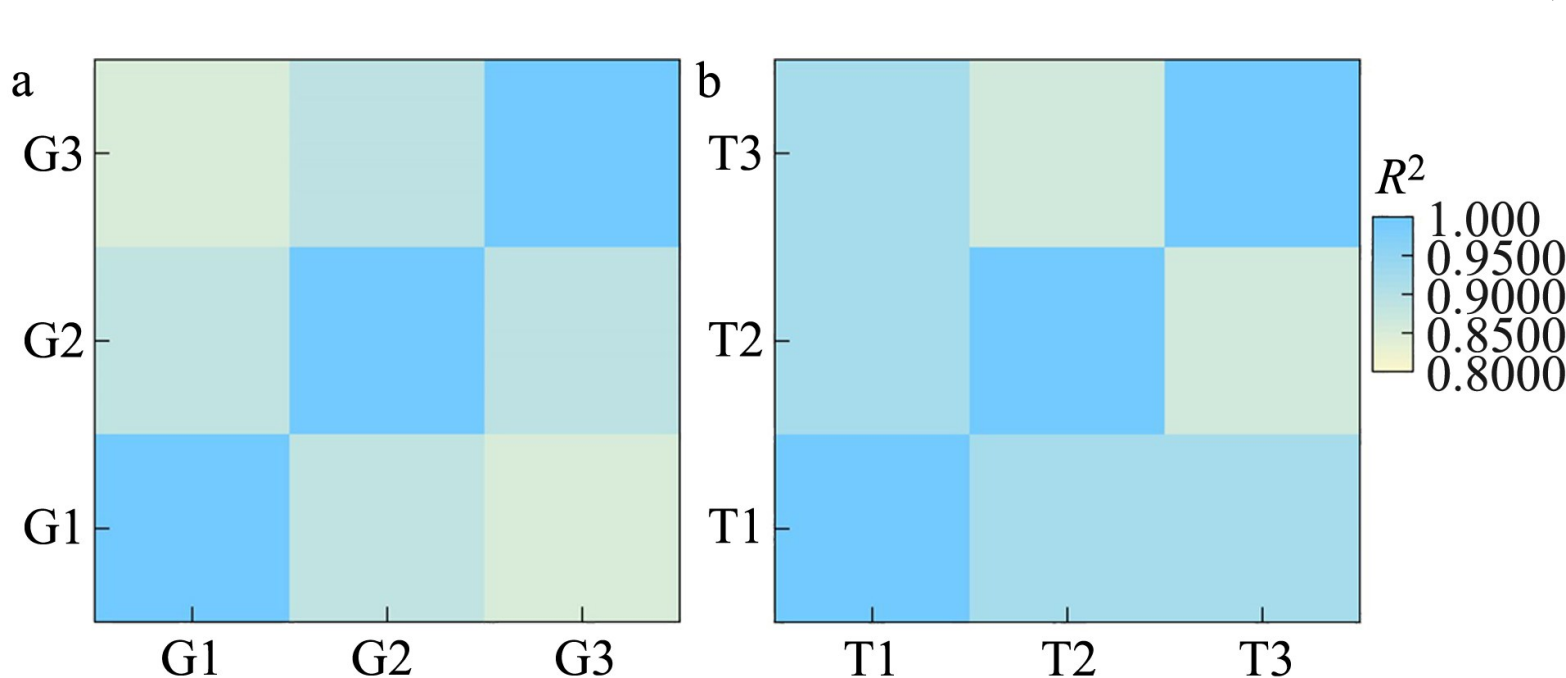
组织切片样品定量到的蛋白质的无标记定量强度分析的重复性

**图7:**
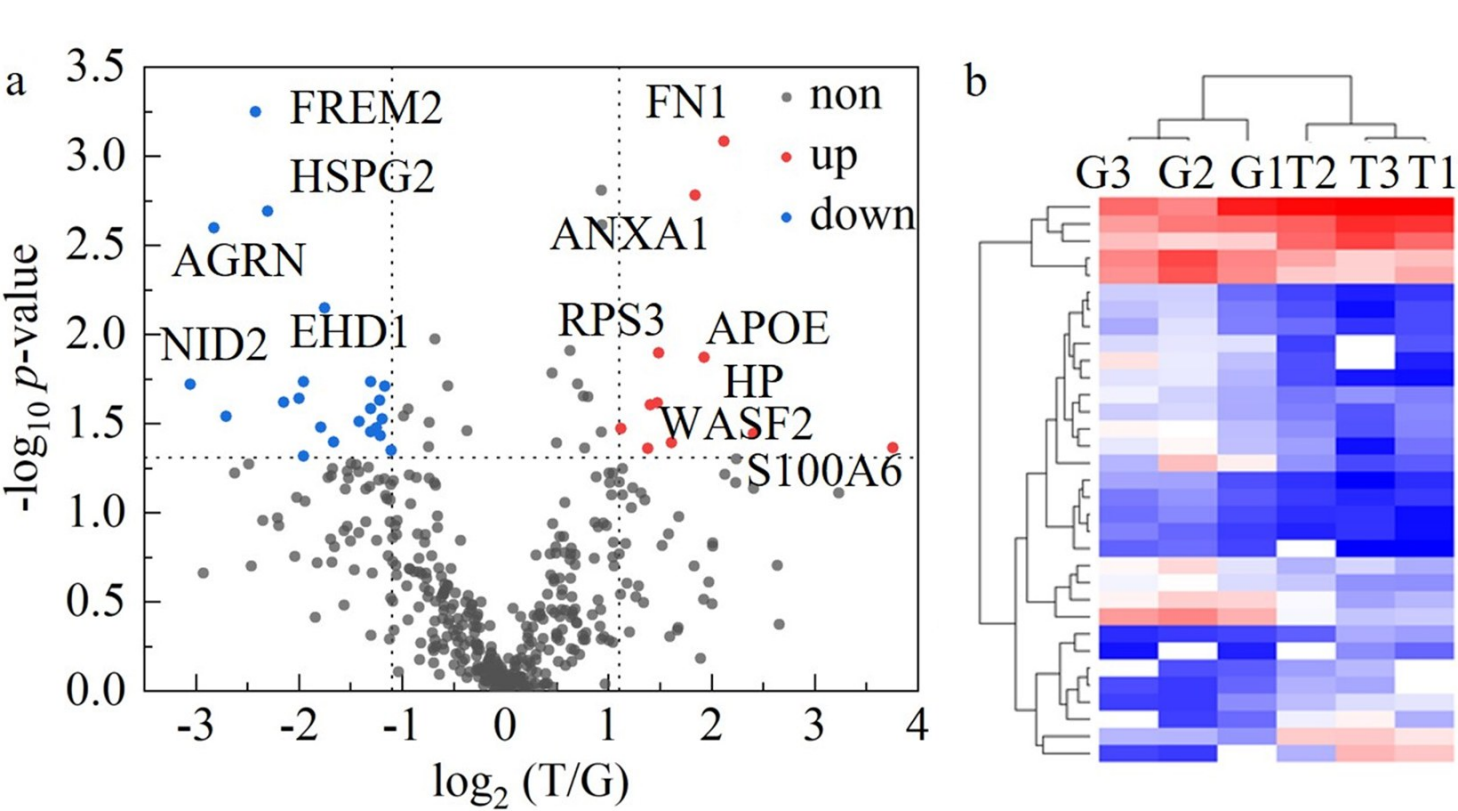
(a)甲状腺肿和甲状腺乳头状癌组织切片样品定量到的蛋白质的火山图和(b)差异蛋白质分级聚类分析图

对不同样品间的差异蛋白质涉及的生物学功能及KEGG通路进行分析。如图8所示,与甲状腺肿样品相比,甲状腺乳头状癌患者明显变化的蛋白质主要参与了细胞外基质受体相互作用(显著性水平*p*<3.4×10^-4^)、对活性氧响应(*p*<2.5×10^-5^)、抗氧化活性(*p*<6.5×10^-4^)、氧化应激反应(*p*<1.2×10^-3^)等过程,而文献报道这些过程与甲状腺疾病患者的代谢过程异常密切相关^[13,14]^,证明此方法可实现高可信度的甲状腺组织切片样品的蛋白质组分析。

**图8:**
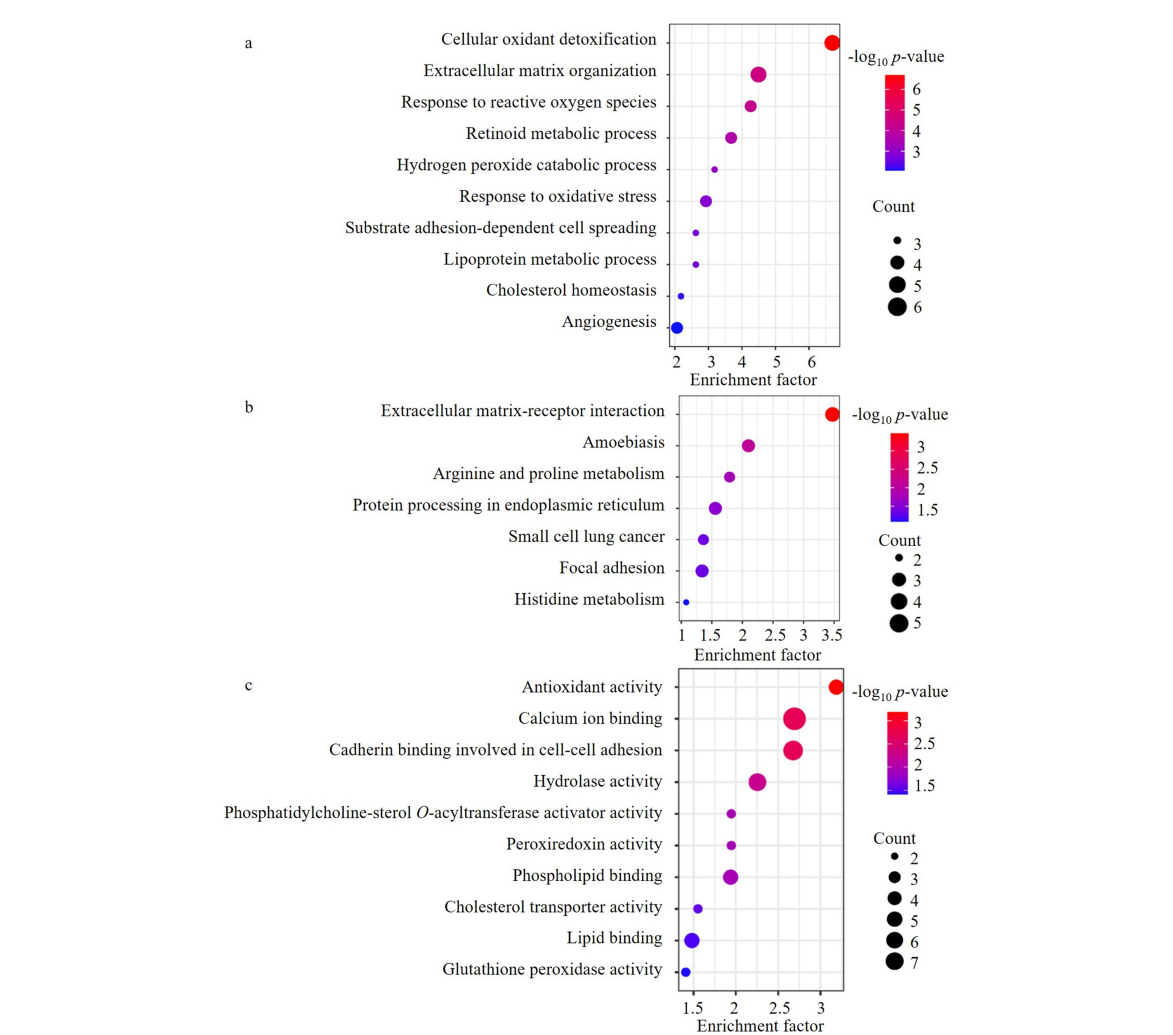
甲状腺肿和甲状腺乳头状癌组织切片样品差异蛋白质的基因本体论(a)生物学过程、(b)KEGG通路和(c)分子学功能分析

对差异蛋白质进一步分析,发现高达57.6%的蛋白质(EHD1、NID2、AGRN、TPO、VCP、LAMC1、RAP1B、RPN2、PDIA3、PRDX5、PRDX6、GSTP1、APOA1、LMNA、ANXA1、APOE、FN1、HP、S100A6)被报道和甲状腺癌及甲状腺相关疾病密切相关^[15⇓⇓⇓-19]^,表明我们的结果与文献报道有较好的一致性。在差异蛋白质中,EH结构域蛋白(Q9H4M9和EHD1)在甲状腺乳头状癌患者中有3.37倍的下调(*p*=0.0071),文献报道此蛋白质在甲状腺癌中扮演着重要的角色,与肿瘤的大小、淋巴结转移和癌症表面生长因子受体(EGFR)的表达紧密相关^[15]^。S100钙结合蛋白A6(P06703和S100A6)在甲状腺乳头状癌患者中有13.6倍的上调变化(*p*=0.043),此蛋白质被报道能够促进癌细胞增值^[16]^,能被用作甲状腺癌治疗的潜在作用靶点。此外,尽管其他的差异表达蛋白质(FREM2、HSPG2、GANAB、CKB、LAMA5、HNRNPK、CNDP2、PAFAH1B2、RAB7A、RO60、MAOA、RPL13A、RPS3、WASF2)在甲状腺乳头状癌中尚未被报道,但已有研究表明它们与卵巢癌、结肠癌、胰腺癌、前列腺癌等疾病密切相关^[20⇓⇓-23]^,这表明它们可能在癌症的发展过程中扮演着关键的指示角色。因此,这些蛋白质作为甲状腺乳头状癌潜在生物标志物的可能性值得进一步研究和探索。可见,基于组织切片蛋白质组的分析在甲状腺乳头状癌潜在生物标志物的发现上具有巨大的应用潜力。

## 3 实验的组织实施及教学反思

### 3.1 实验的组织实施

开放实验课程以基于UHPLC-MS/MS的甲状腺癌组织切片蛋白质组定量分析为题,涉及液相色谱-高分辨质谱仪操作培训、蛋白质组样品制备、液相色谱-质谱分析方法优化等内容。该课程每年开展两次,每次择优遴选学生2~4人,与教师合作共同完成实验项目。本课程采取理论与实验相结合的教学方式,课程安排共32学时,其中理论课程为4学时,实验课程为28学时。理论课程部分包括:液相色谱-质谱联用仪的发展历史(1学时)、仪器结构(2学时)、工作原理(1学时)。实验部分包括:液相色谱-质谱联用仪的操作(6学时)、仪器相关参数优化(4学时)、蛋白质组样品制备方法开发(12学时)、蛋白质组数据的检索与分析(6学时)等。

在课程设计时,教师对学生的学科背景和学习基础进行详细分析。在理论课程的讲授中,教师采用动画、视频等多媒体工具,将复杂的技术原理以直观、生动的方式呈现出来。此外,通过融入历史脉络、理论推导以及实际应用案例,深入剖析技术环节背后的科学逻辑,助力学生构建全面而系统的知识框架。实验课程开始前,教师准备好实验材料和设备,并对学生进行实验安全培训。学生仔细阅读实验指导书,了解实验原理、目的和注意事项。实验过程中,鼓励学生积极思考并自主设计实验,规范记录实验数据、现象和结果。教师在场指导,及时解答问题。实验结束后,学生需整理实验数据并进行成果展示,介绍实验过程、结果、心得体会,思考课程涉及领域的未来研究趋势和跨学科融合的可能性。教师对学生的实验报告和成果展示进行评价,给出成绩和反馈意见。同时,征求学生对课程设置、教学方法和实验指导等方面的意见和建议,以不断优化和完善课程体系。

### 3.2 教学反思

在教学过程中,教师遵循“以学生为主体”的原则,注重与学生的交流互动。学生们在实验过程中能够主动思考、独立解决问题,展现出较强的团队协作精神。通过开放实验课程的学习,学生的创新思维、动手能力和科学素养得到了显著提升。在开放实验课程的教学过程中,仍有一些值得思考和注意的事项。

首先,理论课程内容的设计应该与分析化学和仪器分析理论课程部分的学习紧密连接并适度延伸,以巩固学生基础知识,加深对理论内容的认识。其次,本实验课程中,样品制备时间长且需要连续操作,课程开始前应该与组内每名学生协调好时间再安排实验。同时,在实验间隙组织学生进行文献阅读和实验研讨等活动,提高学生学习的主动性。实验过程中,每位学生的学科背景及基础不同,作为教师应该充分考虑学生的差异性和需求,小组内合理分工协调,以保证实验的顺利进行。在液相色谱-质谱联用仪的操作培训时,学生不敢动手操作仪器,理论知识与实际应用脱节。教师在教学过程中,让学生多思考,勤动手,敢于直面问题并想解决方案。最后,在学习成果展示与交流环节,可以进一步加强互动和合作的氛围。考虑引入同行评议或评委评审的方式,以提供更多的反馈,启发学生进行思考。

## 4 结语

本开放性实验课程充分利用校级公共实验平台优质资源条件,让学生参与并完成实验项目。该课程内容丰富,具备较强的综合性与实用性。课程涵盖了系统的理论教学、大型仪器设备操作培训和前沿的开放性实验项目,充分体现了理论知识与实践技能并重的教育理念。该课程引入液相色谱-质谱仪器操作教学,使学生深入理解色谱质谱的工作原理、掌握大型仪器操作流程和维护要点,提高学生的动手能力及解决问题的能力。此外,本课程设计了基于UHPLC-MS/MS的甲状腺癌组织切片蛋白质组定量分析的开放性实验项目,涉及从蛋白质组样品制备到数据分析全部流程,有利于培养学生的实践能力、创新精神和团队协作能力,提升学生的科研素养,为其未来的科研工作及培养能够适应新时代要求的高水平创新型人才奠定基础。随着教育模式的不断演进,我们期待本课程能够为未来的教育改革提供宝贵的经验和启示。
